# Screening of Undiagnosed Hypothyroidism in Elderly Persons with Diabetes according to Age-Specific Reference Intervals for Serum Thyroid Stimulating Hormone and the Impact of Antidiabetes Drugs

**DOI:** 10.1155/2016/1417408

**Published:** 2016-06-15

**Authors:** Rosita Fontes, Patricia de Fatima dos Santos Teixeira, Mario Vaisman

**Affiliations:** ^1^Hospital Clementino Fraga Filho, Universidade Federal do Rio de Janeiro, 20241260 Rio de Janeiro, RJ, Brazil; ^2^Diagnosticos da America SA, Rio de Janeiro, RJ, Brazil

## Abstract

*Background*. Studies have suggested that hypothyroidism is more frequent in the elderly with diabetes mellitus. However, an adaptation of TSH levels to age should be considered in this assessment. Some antidiabetes drugs reportedly interfere with TSH levels. The objectives of this study were to evaluate the prevalence of undiagnosed hypothyroidism in patients with diabetes and the influence of antidiabetes drugs.* Material and Methods*. 1160 subjects, 60 years and older (751 with diabetes), were studied; results were compared according to diabetes treatment and with persons without diabetes. TSH, FT4, antithyroperoxidase, fasting glucose, and HbA1c were measured.* Results and Discussion*. 6.4% of patients with diabetes had hypothyroidism, a higher prevalence compared with persons without diabetes (5.1%), but lower than observed in many studies. The use of age-specific TSH reference interval (RI) could explain this difference. Patients taking metformin (MTF) had TSH (showed in medians) slightly lower (2.8 mU/L) than those not on MTF (3.3 mU/L), *p* < 0.05. MTF doses influenced TSH levels.* Conclusions*. The use of specific TSH RI could avoid the misdiagnosis of hypothyroidism in elderly with diabetes. Patients in use of MTF as single drug had lower TSH than those using other medications and persons without diabetes.

## 1. Introduction

The occurrence of hypothyroidism in patients with diabetes mellitus has attracted attention since Joslin et al. revealed this association [1]. Several studies have reported a higher prevalence of thyroid dysfunction in diabetes [2–4]. This association may be related to autoimmunity as well as other factors associated with either type 1 or type 2 diabetes and suggests that persons with diabetes be screened for hypothyroidism [2, 5–7]. The Executive Summary of Standards of Medical Care in Diabetes (2014) published by the American Diabetes Association (ADA) [8] as well as The Clinical Practice Guidelines for Hypothyroidism in Adults (2012) copublished by the American Association of Clinical Endocrinologists (AACE) and the American Thyroid Association (ATA) [9] suggest screening patients with type 1 diabetes for thyroid diseases.

Age may be a factor associated with the higher prevalence of hypothyroidism among patients with type 2 diabetes [4, 10]. In elderly patients, however, it may be difficult to determine if an abnormal thyroid stimulating hormone (TSH) pattern is due to actual thyroid disease or an unrelated cause or possibly is secondary to drug interference. Although some studies investigated this, it was not always taken into consideration that the upper reference range for TSH in elderly individuals may be higher than in the general population [10–12]. It is now clear that screening for thyroid dysfunction in elderly subjects with diabetes should take into account that an increase in serum TSH levels is expected.

Previously, an increase in TSH levels in elderly persons was attributed to elevated levels of circulating thyroid antibodies in this specific population. However several recent studies demonstrated that, even after excluding those with known thyroid diseases and circulating antithyroid antibodies, the TSH distribution curve remained shifted to the right; the 97.5th percentile TSH values rose as the population aged [9, 13–17].

When considering screening for thyroid diseases in patients with diabetes, the diverse types of drugs used by a significant number of these patients ought to be taken into account, as many reportedly interfere with TSH levels, especially metformin and sulfonylureas [18–22].

The major aim of this study was to evaluate the prevalence of previously undiagnosed cases of hypothyroidism in the elderly with diabetes by applying specific RI for serum TSH. Furthermore, we evaluate the influence of several antidiabetes drugs on serum TSH.

## 2. Materials and Methods

### 2.1. Patients

A cross-sectional study of 1160 subjects over 60 years of age was performed, of whom 751 had diabetes. All participants underwent routine thyroid function tests, especially as thyroid function or autoimmunity tests had not been performed for all patients previously. The mean time since diabetes diagnosis was 10.2 years (range: 2.6–14.1 years). Only patients with diabetes who had been diagnosed at least 2 years priorly were included; diagnosis was according to the ADA criteria [8]. The blood counts as well as renal and liver functions of all patients were normal. Subjects normally resided in the metropolitan area of Rio de Janeiro and self-identified as belonging to middle and upper social classes. As for race, 68% were white, 20% were mulatto, and 12% were black. Urinary iodine assessment was not performed, since salt iodization in Brazil is determined by federal law [23] and a previous study showed sufficient iodine intake in the Rio de Janeiro population [24]. A National Health Surveillance Agency review of salt from samples used in the city a year before the beginning of our study confirmed appropriate iodine levels [25]. Exclusion criteria were patients under treatment for thyroid diseases or with a history of thyroid disease, use of medications known to interfere with measurement of TSH or free thyroxine (FT4) in the previous three months (except for diabetes treatments), use of contrast media and other medications containing iodine in the last six months, illness, accident, or surgery in the last six months, and chronic disease except for hypertension and dyslipidaemia. There were 409 patients without diabetes who attended a clinical laboratory to collect routine tests for which function and/or autoimmunity thyroid evaluation had not been requested and that had normal blood count, kidney, and liver functions. All of them fulfilled the same exclusion criteria of patients with diabetes and were not under antidiabetes drugs for any other reason.

This study was approved by the institutional review board of Hospital Clementino Fraga Filho, Universidade Federal do Rio de Janeiro. Written informed consent was obtained from all patients.

### 2.2. Tests and Analysis

The tests were performed under the same conditions for both groups. Patients with diabetes were divided into subgroups according to the medications they used as follows: subgroup MTF: metformin (500–3000 mg/day); subgroup SU: sulfonylureas (glibenclamide 5–15 mg/day or glimepiride 1–4 mg/day); subgroup DPP4: dipeptidyl peptidase 4 (DPP4) inhibitors (vildagliptin or sitagliptin, 50–100 mg/day); subgroup TZD: thiazolidinedione (pioglitazone, 15–45 mg/day); subgroup INS: insulin analogues glargine or detemir 13–42 UI, plus analogues lispro or aspart 6–14 UI/day; subgroup MTF+: metformin in combination with one or more other medications (sulfonylurea, DPP4 inhibitor, thiazolidinedione, or insulin analogues); the MTF+ group used lower doses of some medications compared to the doses when they were used as the sole medication: MTF: 500–1500; SU (glibenclamide: 5–10 mg/day, glimepiride: 1-2 mg/day); TZD: 15–30 mg/day; DPP4 and INS doses did not differ from the previous subgroups. All patients with diabetes had been on the same medication for at least one year. Other antidiabetes drugs were not evaluated in the study, as there was an insufficient number of patients to allow statistical analysis.

A questionnaire was administered through direct interviewing to obtain personal and family history data. Body mass index (BMI) was obtained by dividing the body weight (kg) over the square of the height (m^2^). TSH, FT4, antithyroperoxidase antibodies (TPOAb), fasting glucose (FG), and glycated hemoglobin (HbA1c) were measured in all patients.

### 2.3. Biochemical Data

The measurements of the analytes were done on the same day. Serum TSH, FT4, and TPOAb were measured by electrochemiluminescence immunoassays using the Roche Modular Analytics® E170 instrument (Roche Diagnostics Australia Pty Ltd., Castle Hill, NSW, Australia). Serum TSH concentrations were measured by an immunometric method, with an intra-assay percent coefficient of variation (% CV) of 3.0% at concentrations of 0.040 ± 0.001 mU/L, 2.7% at 0.092 ± 0.002 mU/L, and 1.1% at 9.4 ± 0.1 mU/L. The TSH reference interval (RI) established in our previous study of the population in the same region was 0.4 to 5.8 mU/L for subjects aged between 60 and 79 years and 0.4 to 6.7 mU/L for those aged 80 years and older [26]. Serum FT4 and TPOAb were measured by competitive assays. For FT4, the % CV was 1.4% at concentrations of 9.0 ± 0.1 pmol/L (0.7 ± 0.01 ng/dL), 1.8% at 16.7 ± 0.3 pmol/L (1.3 ± 0.02 ng/dL), and 2.0% at 34.7 ± 1.3 pmol/L (2.7 ± 0.1 ng/dL). The RI for age groups, 60 years and older, was in the range of 9.0–21.9 pmol/L (0.7–1.7 ng/dL) [26]. The intra-assay % CV for TPOAb was 6.3% at concentrations of 21.3 ± 1.34 IU/mL, 5.1% at 51.2 ± 2.6 IU/mL, and 2.7% at 473 ± 12.7 IU/mL; a score lower than 34 IU/mL indicated the absence of thyroid disease according to the assay's manufacturer. FG was measured by an enzymatic reference method with hexokinase using Roche/Hitachi Cobas c, GLUC3 (Roche Diagnostics GmbH, Mannheim, Germany). The lower limit of assay detection is 0.1 mmol/L (2.0 mg/dL), with an intra-assay % CV of 0.8% at concentrations of 5.2 ± 0.04 mmol/L (93.2 ± 0.7 mg/dL), 0.7% at 13.4 ± 0.11 mmol/L (241.0 ± 2.0 mg/dL), and 0.7% at 36.1 ± 0.28 mmol/L (651.0 ± 5.0 mg/dL). According to the ADA, the normal value is less than 5.6 mmol/L (100.0 mg/dL) [27]. HbA1c was measured by high-performance liquid chromatography (HPLC) on the Bio-Rad VARIANT II Hemoglobin A1c system (Bio-Rad Laboratories, Inc., Hercules, California). The HbA1c reportable range is between 3.1 and 18.5 percent (%) in National Glycohemoglobin Standardization Program (NGSP) units and between 10 and 179 mmol HbA1c/mol Hb (mmol/mol) in International Federation of Clinical Chemistry and Laboratory Medicine (IFCC) units. The Master Equation for Designated Comparison Method is as follows: IFCC = (10.93 NGSP) − 23.50 (http://www.ngsp.org/convert1.asp). According to the manufacturer, the intra-assay CV was 0.9% for concentrations in healthy subjects and 0.59% for measurements obtained from individuals with diabetes. A patient with diabetes with HbA1c levels below 7.0% (53 mmol/mol IFCC) is sufficiently controlled according to ADA [8].

Hypothyroidism was classified as subclinical if TSH levels were above 5.8 mU/L in patients aged 60–79 years and above 6.7 mU/L in patients aged 80 years or older, with FT4 levels ranging between 9.0 and 21.9 pmol/L (0.7 and 1.7 ng/dL); overt hypothyroidism was diagnosed with the same values of TSH and with serum FT4 below 9.0 pmol/L (0.7 ng/dL) [26]. Autoimmunity was considered present when TPOAb titres were greater than 34 IU/mL.

### 2.4. Statistical Analysis

General data were analyzed using the GraphPad Prism® software, version 6.0 (GraphPad Software, Inc., California). Kolmogorov-Smirnov tests were performed to assess the normal distribution. Log 10 transformations of nonnormal variables were performed for analysis. To test if patients with diabetes and persons without diabetes ages were matched, the unpaired t-test with Welch's correction was used, as age had normal distribution. Descriptive analyses of serum TSH, FG, and HbA1C were reported as medians and 25 and 75 percentiles, because they were not normally distributed. Descriptive analysis of serum FT4 was reported as mean with standard deviation, as it was normally distributed. Two tailed Mann-Whitney and Kruskal-Wallis tests were used to compare the nonparametric TSH distributions in different subpopulations. Comparison between two TSH subgroups was performed by an independent test of Dunn multiple comparisons. Bonferroni correction was applied to ensure that the results were more consistent. For all parameters, *p* < 0.05 was considered statistically significant.

To describe the relationship between TSH (mU/L) and MTF dose (mg/day), a statistical model was used assessing the possible significance of MTF effect in the TSH variability and providing estimated values for TSH according to different doses. The class of generalized linear models (GLM) was used to build the model, since the TSH distribution was not normal. Likelihood-ratio tests were used to assess the significance of parameters estimates, and half-normal plots with simulation envelopes were used to assess goodness-of-fit of the models. The analysis was performed using R software (R Core Team, 2014), and the R package hnp was used to the half-normal plots. In addition, predicted and average curves were compared and residuals were analyzed.

## 3. Results

Data of parameters not normally distributed are shown as follows: median, 25–75 percentiles (95% CI). Those normally distributed are shown as mean ± SD. There were no statistically significant differences in the mean of age between patients with diabetes and persons without diabetes, so that both groups were matching for study according to the age. The baseline demographic, clinical, and general laboratory data are presented in Table 1. Data of TSH, FT4, and autoimmunity in euthyroid patients are in Table 2, as well as the occurrence of hypothyroidism and autoimmunity in hypothyroid patients.

A lower rate of circulating antibody among hypothyroid patients was observed in the subgroup of pioglitazone [25% (1/4)]; however, the small number in the group did not justify a statistical analysis.

In relation to hormonal data between antidiabetic drugs subgroups, only MTF and INS showed significantly different results from the others. Thus SU, DPP4, and TZD were analyzed together. MTF+ subgroup is shown separately, since we had interest in checking whether this subgroup behaved as the subgroup only on MTF or as the other subgroups, since the MTF doses used were lower.  Table 3 shows the results of euthyroid patients and Table 4 those of hypothyroid patients.

When we compared clinical and subclinical hypothyroidism between patients with diabetes as a whole group and persons without diabetes, the results in patients with clinical hypothyroidism were as follows: TSH values: 17.5, 13.0–17.3 (95% CI 12.2–16.4) mU/L in patients with diabetes and 15.6, 13.4–16.8 (95% CI 11.9–16.1) mU/L in persons without diabetes (*p* = NS); FT4 values: 7.1 ± 0.3 (95% CI 6.6–7.6) pmol/L [0.55 ± 0.02 (95% CI 0.51–0.59) ng/dL] and 7.7 ± 0.1 (95% CI 6.8–7.9) pmol/L [0.6 ± 0.01 (95% CI 0.53–0.61) ng/dL], respectively (*p* = NS). For patients with subclinical hypothyroidism TSH values were 12.3, 10.4–14.0 (95% CI 11.2–14.8) mU/L in patients with diabetes and 10.9, 9.4–13.8, (95% CI 10.7–13.7) mU/L in persons without diabetes, with a marginal significance (*p* = 0.049); and FT4 values were 14.2 ± 0.39 (95% CI 11.5–12.0) pmol/L [1.1 ± 0.03 (95% CI 0.89–0.93) ng/dL] and 14.2 ± 0.3 (95% CI 11.8–14.2) pmol/L [1.1 ± 0.02 (95% CI 0.92–1.1) ng/dL], respectively, *p* = NS.

**Figure 1:**
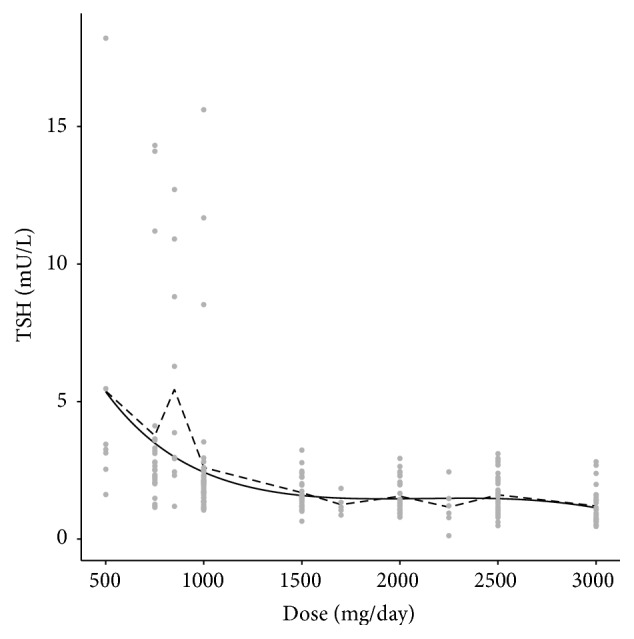
Estimated TSH according to the MTF dose. Comparison between observed and predicted values. Points represent each patient, the dashed line represents the observed average TSH, and the full line represents the estimated average TSH.

In addition to the data cited, it is noteworthy that TSH in MTF users was also lower than in persons without diabetes (*p* = 0.032). INS compared to all other subgroups together had highest levels of FG, HbA1c, and BMI. These parameters were as follows (MTF × others, resp.): FG: 8.4, 6.6–10.6 (95% CI, 7.3–10.4) × 7.3, 5.6–9.3 (95% CI 6.5–8.8), *p* = 0.004; HbA1c: 7.6, 6.4–9.0, (95% CI 5.8–10.7) × 7.1, 6.2–8.9 (95% CI 6.8–9.2), *p* = 0.0381; and BMI: 30.9 ± 6.3 × 28.4 ± 5.2, *p* = 0.0061. With regard to the relationship between TSH levels and MTF dose the selected model assumes an inverse distribution for the square root of TSH in the random component of the GLM, an identity link function, and a systematic component (linear predictor) with intercept and terms until third order to the dose of metformin. Symbolically, we have the estimated TSH with MTF dose varying from 500 to 3000 mg/day. Parameters estimates and standard errors, according to likelihood-ratio tests, were all significant. The half-normal plot related to this model indicates a good fit, since less than 5% of the points fell out of the simulation envelope. Even more, the residuals have mean, first, and third quartiles close to zero: −0.0017, −0.2540, and 0.1398, respectively. A graphical representation of the model is presented in Figure 1, showing that the two curves are close. The overall conclusion is that there was a significant reduction of TSH as the MTF dosage increased.

**Figure 2:**
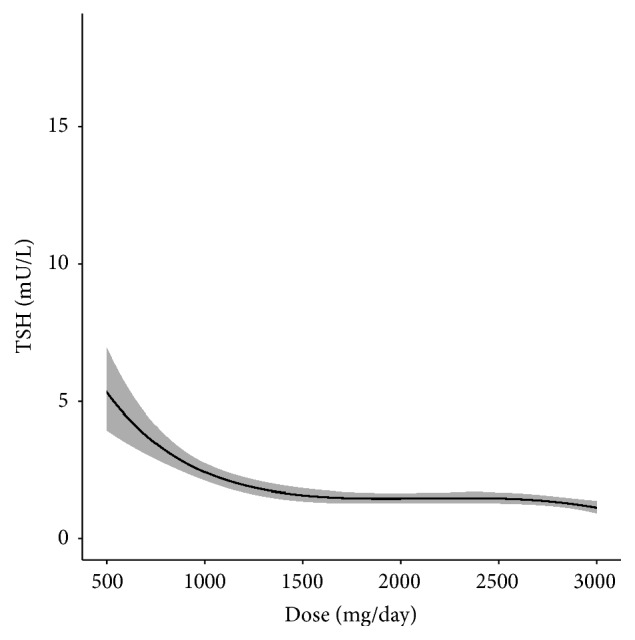
95% confidence interval for the estimated TSH according to the MTF dose.

It was also possible to obtain a confidence interval for the estimated TSH, based on the asymptotic properties of the maximum likelihood estimators.  Figure 2 shows the 95% confidence interval.

Regarding a possible influence of MTF+ subgroup on the TSH, this was not observed in the same model, and therefore, the curves were not performed.

In order to assess whether an age-specific TSH RI to older patients had a clinical impact with respect to the diagnosis of hypothyroidism in patients with diabetes, we compared the amount of hypothyroidism diagnosis in these patients when using TSH adjusted for age with the amount that would be diagnosed if using the RI defined by the kit manufacturer. In the first case 6.4% (45/751) were diagnosed as hypothyroid patients with diabetes. This diagnosis was increased to 14.6% (110/751), that is, more than twice, if the RI were adopted without discrimination by age.

## 4. Discussion

In this study, we aimed to evaluate the prevalence of undiagnosed hypothyroidism in patients with diabetes over 60 years of age by adopting age-specific RI. Using this method, we observed that the diagnosis of hypothyroidism was slightly but significantly higher in patients with diabetes than in persons without diabetes, albeit less frequent than reported in some previous studies. A PubMed search revealed that most studies on the prevalence of hypothyroidism in patients with diabetes were conducted before recent revelations in the field about TSH values in the elderly. This rate is quite variable, ranging from 5.7% to 27.7% in previous studies [2, 3, 5, 10, 12]. In the patients with diabetes population we studied, undiagnosed cases of hypothyroidism were similar to those that showed lower prevalence. The difference among the various studies could be attributed to which RI were used for the diagnosis of hypothyroidism, to the heterogeneity of patients' ages, including in the same group those younger than 60 years and those older than 60 years, and to the characteristics of each population.

There were higher rates of positive TPOAb in patients with diabetes (13.8%), especially in hypothyroidism patients (64.4%). Of note, the percentage of patients with diabetes with hypothyroidism and TPOAb positivity was higher than that observed in other studies, which ranged from 14.6% to 43% [6, 7, 10]. Autoimmunity directed against the thyroid can vary depending on factors such as patients' ages, environmental factors, and the laboratory methods used [28]. As this study focused on a select population of elderly subjects, this could at least partially explain the higher values. The involvement of environmental factors including differences in iodine diet content, stress, and drugs could be other factors to be considered. The frequency of circulating TPOAb was similar in all groups except group TZD. This is discussed further below.

Although TSH levels were equivalent when comparing patients with diabetes to persons without diabetes overall, patients with diabetes who were given MTF had slightly but significantly lower TSH compared to persons without diabetes as well as to patients with diabetes treated with other antidiabetes agents. MTF is the most widely used drug to treat diabetes and is the first choice medication recommended for the treatment of type 2 diabetes by ADA [29]. Results of previous studies of the effect of MTF on TSH levels are controversial. Some reports suggest that all subjects treated with MTF likely have lower TSH levels [30, 31], while others report no significant lowering of TSH by the drug, except in those subjects with subclinical hypothyroidism or who are on levothyroxine treatment [19, 32].

The reason MTF may reduce TSH may be due to its reported capacity to cross the blood brain barrier. It directly acts in several regions of the brain and can concentrate in the pituitaries and hypothalamuses of rats and can also suppress AMP-activated protein kinase activity (AMPK) [33, 34]. As hypothalamic triiodothyronine administration also decreases this enzyme, [35] it is postulated that MTF could decrease TSH in the same manner as thyroid hormone, but to a lesser degree. This idea is further supported by a study that suggests that MTF may have an impact on thyrotrope function in hypothyroid patients, lowering TSH in polycystic ovary syndrome, which was attributed to a probable effect increasing thyroid hormone action in the pituitary [36]. We observed an inverse correlation between doses of MTF and TSH. When evaluating patients being given a combination of MTF and one of the other drugs, lower TSH levels were not significant. A possible explanation is that the MTF doses in MTF+ subgroup were smaller than those on MTF as a sole medication. This could be the reason TSH have not been affected by MTF in combination with other antidiabetic drugs. We question whether the relation between TSH and MTF doses could explain the different results observed regarding the effect of this medication on TSH levels when comparing different series. At this point, these results cannot be considered definitive but are preliminary data that offer new perspectives regarding the relationship between MTF and TSH, requiring confirmation with a larger number of cases.

It has been suggested that the effect of MTF on TSH could be secondary to weight reduction. In the model adopted, we did not compare the impacts of each medication on patients' weights, but the BMI of each treatment subgroup was compared with the others. At the time of data collection, all subjects except those on INS treatment had similar BMI. Therefore, weight did not appear to be a significant factor in the effect of MTF on TSH levels compared to other medications [19].

Other antidiabetes medications have been related to TSH changes. Early studies suggested that the first generation of SU could raise TSH levels. Investigators also pointed to a goitrogenic effect as well as a decrease in iodine uptake by the thyroid gland with second-generation SU gliclazide [37]. In our study, glibenclamide or glimepiride did not affect TSH levels when administered with the doses indicated. These effects were also not observed in another study of second-generation SU glyburide [38].

Regarding DPP4, no changes were observed in TSH between this subgroup and either persons without diabetes or subjects in the other diabetes treatment groups. A search of the PubMed database using the terms “DPP4 inhibitors”, “thyroid”, “TSH”, “hypothyroidism”, and “hyperthyroidism” did not yield any studies related to TSH changes with DPP4 inhibitors.

The TSH levels of patients who used pioglitazone (TZD) did not differ from other subgroups. Pioglitazone is a TZD, a class of drugs that act as an exogenous peroxisome proliferator-activated receptor-*γ* (PPAR*γ*) agonist. Besides controlling glucose metabolism and fatty acid storage, PPAR*γ* is strongly expressed in the thyroid tissues of patients with autoimmune thyroid disease. Pioglitazone is also capable of lowering the expression and release of certain cytokines in thyroid cells [39]. We observed that TPOAb levels were lower in TZD subgroup of patients with diabetes; however we could not make conclusions regarding the effect of pioglitazone on autoimmunity because of the small number of patients. Future studies with PPAR*γ* agonists in autoimmune thyroid diseases are necessary to determine any such effect.

Patients using INS had the highest TSH median among the groups. We postulate that this may be due to selection bias because this group also had the highest BMI and HbA1c levels. Previous studies showed a positive correlation between TSH, even within its normal range, and body weight/BMI and HbA1c [12, 40–42]. One study linked increased weight with the susceptibility to harbor thyroid autoimmunity [43]. We did not consider this aspect, as TPOAb values in patients using INS (the subgroup with higher BMIs) were not different when compared to the other subgroups.

Considering the most appropriate evaluation of the prevalence of undiagnosed hypothyroidism among patients with diabetes, especially the elderly, our results justify widening the TSH RI [9] and applying appropriate levels for the geriatric population. Doing so may lead to a reduction in mistaken diagnoses and avoid unnecessary treatments. Possible bias in this study is that of other drug interference, since polymedicine is prevalent in elderly patients; besides that, thyroid function was only measured once and thus cases of subclinical thyroid dysfunction could be due to nondetected nonthyroidal illness during the selection. We attempted to minimize some of these possible biases, by excluding those subjects who used medications that are known to interfere with TSH levels. The authors suggest that an annual TSH measurement in DM patients over 60 years would be appropriate for hypothyroidism screening. If TSH levels do begin to increase at any time in relation to the specific RI for age, FT4 and TPOAb levels should be measured.

In conclusion, in patients with diabetes aged 60 years or older, hypothyroidism was more prevalent than in persons without diabetes of the same age, justifying the suggestion of annual screening in these patients. Use of TSH RI appropriate for the elderly can avoid the misdiagnosis of hypothyroidism. In this study we found an interesting association between the MTF dose when used as a single medication and TSH levels, encouraging that further studies are conducted on this issue.

## Figures and Tables

**Table 1:** Baseline data of all subjects studied: number, age, body mass index, fasting glucose, and glycated hemoglobin in patients with diabetes and persons without diabetes.

Data	Patients with diabetes	Persons without diabetes	*p* values
Males/females (*n*/%)	332 (44.2%)/419 (55.8%)	176 (43.0%)/233 (57.0%)	—

Age (years)	71.3 ± 7.0	69.9 ± 7.6	NS

BMI (kg/m^2^)	28.3 ± 5.3	27.5 ± 5.8	0.044

FG (nmol/L)			
Median	7.6	4.6	0.008
25–75%	5.5–10.6	4.1–5.3	
95% CI	7.2–9.8	4.3–5.6	

HbA1c (% total Hb)			
Median	7.2	5.2	0.0003
25–75%	6.0–9.2	5.1–5.6	
95% CI	6.8–8.8	5.1–5.4	

BMI: body mass index; FG: fasting glucose; HbA1c: glycated haemoglobin; 25–75%: 25–75 percentiles; CI: confidence interval. For converting FG in mg/dL, multiply nmol/L by 18; for converting HbA1c in % to IFCC mmol/mol, use the equation (10.93 × NGSP) − 23.50.

**Table 2:** Laboratory data in euthyroid patients and occurrence of hypothyroidism and autoimmunity in hypothyroid patients with diabetes and persons without diabetes.

Data	Patients with diabetes	Persons without diabetes	*p* values
TSH (mU/L)			
Median	3.3	3.2	NS
25–75%	1.1–4.3	1.3–4.2	
95% CI	3.1–4.7	2.9–3.9	

FT4 (pmol/L)			
Mean	14.2	15.4	NS
SD	±3.9	±2.6	
95% CI	14.5–15.2	14.2–15.4	

Positive TPOAb (%) in euthyroid patients	13.8% (104/751)	8.3% (34/409)	0.027

Hypothyroidism (%/*n*)	6.4% (45/751)	5.1% (21/409)	0.0038

Positive TPOAb (%) in hypothyroid patients	64.4% (29/45)	34% (7/21)	<0.0001

TSH: thyroid stimulating hormone; FT4: free thyroxine; 25–75%: 25–75 percentiles; CI: confidence interval; NS: not significant. For converting FT4 in nd/dL, multiply pmol/L by 0.078.

**Table 3:** TSH and FT4 in subgroups of euthyroid patients with diabetes.

	Subgroups of antidiabetes drugs				*p* values
	MTF	SU/DPP4/TZD	INS	MTF+	
TSH (mU/L)					
Median	2.8	3.3	4.1	3.2	MTF × other groups < 0.05 INS × other groups < 0.05
25–75%	2.4–3.5	2.8–3.9	3.6–5.3	2.4–4.7	
95% CI	2.9–3.4	2.9–3.6	4.4–6.5	3.0–4.3	

FT4 (pmol/L)					
Mean	14.2	14.4	15.4	14.2	NS
SD	±0.5	±0.9	±2.6	±2.6	
95% CI	12.9–16.7	10.3–15.4	12.9–16.7	14.2–15.4	

TSH: thyroid stimulating hormone; FT4: free thyroxine; 25–75%: 25–75 percentiles CI: confidence interval; NS: not significant. MTF: metformin; SU/DPP4/TZD: sulfonylureas, dipeptidyl peptidase 4 (DPP4) inhibitors, and thiazolidinedione analyzed together. For converting FT4 in nd/dL, multiply pmol/L by 0.078.

**Table 4:** Prevalence of hypothyroidism, TSH, and FT4 in subgroups of hypothyroid patients with diabetes.

	Subgroups of antidiabetes drugs				*p* values
	MTF	SU/DPP4/TZD	INS	MTF+	
Hypothyroidism (%/*n*)	4.5% (10/224)	6.5% (14/215)	7.5% (4/53)	6.6% (17/259)	MTF × other groups < 0.05 MTF+ × other groups NS

TSH (mU/L)					
Median	11.8	15.0	17.5	13.5	MTF × other groups < 0.05 INS × other groups < 0.05 MTF+ × other groups NS
25–75%	11.6–14.2	11.2–17.1	13.2–19.3	11.7–15.6	
95% CI	11.5–13.7	14.5–15.5	17.0–18.0	12.9–15.9	

FT4 (pmol/L)					
Mean	11.7	11.1	9.8	10.9	NS
SD	±0.5	±0.5	±1.3	±1.3	
95% CI	10.3–15.4	10.3–15.4	9.0–11.6	9.0–11.6	

TSH: thyroid stimulating hormone; FT4: free thyroxine; 25–75%: 25–75 percentiles CI: confidence interval; NS: not significant. MTF: metformin; SU/DPP4/TZD: sulfonylureas, dipeptidyl peptidase 4 (DPP4) inhibitors, and thiazolidinedione analyzed together. For converting FT4 in nd/dL, multiply pmol/L by 0.078.
